# Network Analysis of Well-Being Dimensions in Vaccinated and Unvaccinated Samples of University Students from Poland during the Fourth Wave of the COVID-19 Pandemic

**DOI:** 10.3390/vaccines10081334

**Published:** 2022-08-17

**Authors:** Aleksandra M. Rogowska, Karolina Chilicka, Dominika Ochnik, Maria Paradowska, Dominika Nowicka, Dawid Bojarski, Maciej Tomasiewicz, Zuzanna Filipowicz, Maksymilian Grabarczyk, Zuzanna Babińska

**Affiliations:** 1 Institute of Psychology, University of Opole, 45-052 Opole, Poland; 2 Department of Health Sciences, University of Opole, 45-040 Opole, Poland; 3 Faculty of Medicine, University of Technology, 40-555 Katowice, Poland; 4 Faculty of Psychology and Cognitive Studies, Adam Mickiewicz University in Poznan, 60-568 Poznan, Poland; 5 Faculty of Sociology, University of Warsaw, 00-927 Warsaw, Poland; 6 Faculty of Medicine, Wroclaw Medical University, 50-367 Wroclaw, Poland; 7 Department of Pharmacology, Medical University of Bialystok, 15-089 Bialystok, Poland; 8 Faculty of Medicine, Poznan University of Medical Sciences, 61-701 Poznan, Poland; 9 Institute of the Middle and the Far East, Faculty of International and Political Studies, Jagiellonian University, 30-063 Krakov, Poland

**Keywords:** mental health, network analysis, physical health, subjective well-being, university students, vaccination intention and decision

## Abstract

Although numerous studies investigated the predictors of vaccination intention and decision, little is known about the relationship between vaccination and well-being. This study compares the physical and mental health dimensions among vaccinated and unvaccinated people. In a cross-sectional online survey, 706 university students from Poland (mean age of 23 years, 76% of women) participated in this study during the fourth pandemic wave (November–December 2021). Standardized questionnaires with a Likert response scale were included in the survey to measure spirituality, exposure to the COVID-19 pandemic, perceived physical health, stress, coronavirus-related PTSD, fear of COVID-19, anxiety, depression, and life satisfaction. Consistent with the fuzzy-trace theory, the unvaccinated sample was younger and scored significantly lower than the vaccinated group in exposure to COVID-19, perceived physical health, stress, coronavirus-related PTSD, fear of COVID-19, and depression, while higher in life satisfaction. The network analysis showed that mental health plays a crucial role in both groups, with the central influence of anxiety and stress on depression and life satisfaction. The message on vaccination to university students should focus on the benefits of vaccination in maintaining the status quo of good health and well-being. Campus prevention programs should primarily aim to reduce anxiety, stress, and negative emotions by teaching students coping strategies, relaxation techniques, and mindfulness.

## 1. Introduction

The COVID-19 pandemic significantly impacted well-being, deteriorating populations’ physical and mental health worldwide. Several restrictions were introduced during the lockdown, such as social distancing and isolation, wearing masks, periodically closing schools, universities, or workplaces, losing jobs, and deteriorating the economic situation of many people [1]. Symptoms of stress, anxiety, and depression significantly increased, while sleep quality and life satisfaction worsened during the successive waves of the pandemic [2,3,4]. Additionally, an increase in unhealthy lifestyles was observed in Italian adults, although they simultaneously presented a good level of knowledge about coronavirus prevention [5]. Women and university students, in particular, were identified as more vulnerable groups during the pandemic [6,7,8,9,10,11,12]. Although COVID-19 vaccines effectively reduce the risk of SARS-CoV-2 infection severity, serious side effects, and death [13,14], some people still remain unvaccinated. For example, among Polish university students, 82% (*n* = 1393) were vaccinated, while 18% (*n* = 303) were unvaccinated during the third wave of the COVID-19 pandemic (March–April 2021) [15]. However, findings indicated that booster vaccinations, physical distancing, and other personal protective equipment could still benefit post-pandemic time [16,17].

Many studies explored the various factors that determine people’s intention and decision about vaccination, including demographic variables, perceived norms, susceptibility and severity, reasons and motives to receive the vaccine, facilitators and barriers of vaccination, self-efficacy, cues to action, political preferences, perceptions, attitudes, knowledge, and contextual features of COVID-19 vaccination [6,18,19,20,21,22,23,24,25,26,27,28,29,30,31,32,33,34,35]. Research showed that, on average, unvaccinated people were younger (75% of the unvaccinated were under age 50), less educated, with low income, generally more economically disadvantaged, from rural areas, without contact with vulnerable people or who tested positive for COVID-19, and less likely to be married than those vaccinated [18,19,21,25,26,32,33,35]. Country and regional differences in their acceptance of vaccines were also found [29,30,32]. Gender seems to be equivocally related to vaccination. In some studies, vaccine hesitancy was higher among women [21,23,25,27,29], whereas in others, among men [22].

Unvaccinated people have reported reasons for not being vaccinated against COVID, such as lack of confidence in the vaccine and its safety, overall ineffectiveness of vaccination, lack of trust in the governmental pandemic management, fear of vaccine adverse side effects (such as harm, infertility, severe disability, and death), and belief in conspiracy theories [19,20,23,24,26,27,28,30,34,35]. Additionally, lower vaccination willingness was related to insufficient knowledge about SARS-CoV-2, social media influence, trust in authoritative information sources, unclear information on the vaccination program, insufficient data on safety and effectiveness, and inappropriate health beliefs [26,33,34].

Furthermore, the relationship between vaccination and health is inconclusive. Some studies found a higher acceptance of vaccination among people having a chronic disease and comorbidities, those with experienced coronavirus infection, and individuals perceiving a high risk and severity of COVID-19 infection [22,27,28,30]. In contrast, the other studies indicated a higher willingness to receive vaccines among those who perceived physical health as good or very good, individuals without a previous COVID-19 diagnosis, and those with increased fear of COVID-19 [23,25]. Lower levels of anxiety, fear of infection, and better quality of life were reported in fully vaccinated Polish participants (several doses) compared with their counterparts awaiting vaccination or those with one vaccination dose [15]. Among the healthcare workers from Bangladesh, lower rates of somatic and mental health problems were found in vaccinated than in unvaccinated individuals, including general health, depression, post-traumatic stress disorder (PTSD), insomnia, and loneliness [6]. Although previously referred studies included some particular mental health or well-being dimension associated with vaccinating behavior, the complex model of associations between various mental health dimensions and vaccination was never explored or fully explained. However, since mental health dimensions and well-being are intercorrelated, it is necessary to consider them in one comprehensive model.

This research aims to explain the relationship between vaccination and the various well-being dimensions of university students during the fourth wave of the COVID-19 pandemic. Most previous research on determinants of vaccination was performed using regression methods. In this study, we implement the network analysis (NA) method, which allows for the exploration of patterns of links between nodes, represented in the current research by selected demographic variables (i.e., age, gender, relationship status, and exposure to COVID-19 pandemic) and well-being dimensions (spirituality, perceived physical health, stress, symptoms of post-traumatic stress disorder, anxiety, and depression). The structure can reveal close and distant relationships and central and peripheral nodes within the network. The NA was previously used in social sciences, medicine, and health sciences areas as a helpful tool, providing insight into the interplay of behavioral, cognitive, and mental health dimensions, which interact with mental or behavioral problems in everyday functioning [36,37]. In health psychology, the NA was broadly used to examine how the complex interactions between social, psychological, and biological factors contribute to physical and mental health and well-being [38,39]. We focused on identifying the most influential factors for vaccinated versus unvaccinated students. The NA allows finding variables essential for the decision to vaccinate, which may have potential clinical implications in targeted intervention, prevention, or promoting actions.

According to the fuzzy-trace theory approach [40], incomplete knowledge affects the inability to distinguish the essential meaning of the information on vaccination. The decision about vaccination is based on selection between two categorical options: (1) maintaining the unvaccinated status quo of “filing okay,” and (2) the decision to receive a vaccine with two possible consequences: “feeling okay” or “not feeling okay” (because of adverse side effects, causing disease, or death). Since “feeling good” is better than “feeling bad,” many people decide not to receive the vaccine. The decision to vaccinate is based on knowledge, experience, and prior beliefs. Consistent with the fuzzy-trace theory, we expect higher rates of unvaccinated people among university students with good overall physical and mental health and those with a heightened subjective sense of well-being in the current study. For the first time, to our best knowledge, the fuzzy-trace theory [40] is examined during the COVID-19 pandemic. Since university students are the most vulnerable population at risk of mental health issues, they were considered the target sample in the present research.

## 2. Materials and Methods

### 2.1. Study Design and Procedure

Using the snowball sampling technique, a cross-sectional study was conducted between 25 November and 27 December 2021, during the fourth wave of the COVID-19 pandemic in Poland. The online survey (Google Forms) included informed consent, sociodemographic questions, and standardized questionnaires with possible response options to choose from. Surveys were distributed by academic staff via educational e-learning platforms (such as Moodle and Teams) and using a personal e-mailing list. Additionally, surveys were disseminated by university students to their friends and various private and open access students’ groups on social media (such as Facebook and Instagram), namely “Psychologia UAM” “Psychologia AMU”, “Ethnologia AMU”, “Studenci AMU 2020”, “Studenci WPiK AMU”, “Studenckie Radio Żak”, “PSSiAP Poznań”, “Ogólnopolska grupa dyskusyjna dla studentów”, “Uniwersytet Medyczny w Warszawie 2018–2024”, “MISH UW”, “Grupa dyskusyjna Polskiej Partii Socialistycznej”, “Historia UW”, “Peer Support IFMSA Poland”, “Pharmaceuticals UMB 2019–2024”, “PTSF Białystok”, and “Studenci UMB”. Students were recruited from University of Opole, the University of Technology in Katowice, Jagiellonian University, University of Warsaw, Poznan University of Medical Sciences, Białystok Medical University, Wroclaw Medical University, Maria Curie-Sklodowska University in Lublin, Lodz University of Technology, Nicolaus Copernicus University in Toruń, PSWPS University, University of Lodz, Opole University of Technology, and University of Zielona Gora.

The inclusion criteria were an age equal to or above 18 and to be a university student. All respondents were eligible for inclusion in the research and confirmed their student status by answering their age, faculty, major, level, year, and type of study. Over 1215 thousand people studied in the 2020/21 academic year at 349 universities in Poland. The required sample size for network analysis is 500 observations [37]. Initially, 717 people answered the invitation to the survey, but 11 people refused to participate, so the final sample consisted of 706 university students (98% response rate).

The study was anonymous and confidential. Informed consent was obtained from all the students who participated in the survey since information about the study was presented on the first webpage. The University Research Ethics Committee at the University of Opole (Decision No. 1, 22 April 2020) approved the study protocol according to the principles of the Declaration of Helsinki. Participation in the study was not financially compensated.

### 2.2. Measures

The authors developed a questionnaire regarding the students’ exposure to the COVID-19 pandemic following the study of Tang et al. [41]. Participants responded (*Yes* = 1, *No* = 0) to eight categorical questions: (1) Have you experienced symptoms that could indicate coronavirus infection? (2) Have you been tested for coronavirus? (3) did coronavirus hospitalize you? (4) Did you have to be in strict quarantine for at least 14 days and isolated from loved ones because of the coronavirus infection? (5) Has anyone in your family or friend group been infected with coronavirus? (6) Have any of your relatives died of coronavirus? (7) Have you or a loved one lost their job because of coronavirus? (8) Are you currently experiencing a worsening of your functioning or economic status due to the coronavirus pandemic? All eight items were summarized to a composite score, indicating the degree of exposure to the COVID-19 pandemic. The ordinal reliability in this study was α = 0.75. Intelligibility was assessed by six experts in health and stress psychology, using the content validity index CVI = 85.13.

Spirituality was assessed on a 4-point Likert-type scale (from *Not at all spiritual/religious* = 0, to *Very spiritual/religious* = 3) as a response to two questions: (1) “How spiritual do you consider yourself to be?”; and (2) “How religious do you consider yourself to be?” The authors developed these questions based on the Baylor Religion Survey [42]. Higher scores mean higher self-reported spirituality. The reliability for the two-item scale was Cronbach’s α = 79 in this study. The CVI = 91.50 was rated by six experts in health psychology and the psychology of religiosity and spirituality.

Physical health was measured by two items of the General Self-Rated Health (GSRH) [43]. The scale consists of 2 items from the 12-item Short Form survey (SF-12). Individuals rated their health on the 5-point Likert scale (from *Excellent* = 1 to *Poor* = 5). Poor health is related to higher scores, while good health is associated with lower levels. The reliability was Cronbach’s α = 0.87 in this study.

Perceived stress was assessed using the 10-item Perceived Stress Scale (PSS-10) [44]. Participants rated the frequency of their experience of stressful events during the past month on a five-point Likert scale (from *Never* = 0, to *Very often* = 4). The higher the perceived stress, the greater the scores. Cronbach’s α was 0.88 in this study.

Coronavirus-related post-traumatic stress disorder (PTSD) symptoms were measured using the adapted version of the short 6-item PTSD Checklist (PCL-6) [45,46,47]. Answers to six questions about the severity of stressful experiences related to the COVID-19 pandemic in the past month were selected from a 5-point Likert scale (from *Not at all* = 1, to *Extremely* = 5). Higher scores indicate a higher risk of PTSD. Cronbach’s α was 0.76 in this sample.

Fear of coronavirus was assessed using the Fear of COVID-19 Scale (FCV-19S) [48]. Participants answered seven items, rating their current emotional and physiological responses to the COVID-19 pandemic on a 5-point Likert scale (from *Strongly disagree* = 1 to *Strongly agree* = 5). A higher score is interpreted as a greater fear of COVID-19. Reliability in this study sample was Cronbach’s α = 0.85.

Anxiety symptoms were measured using the 7-item Generalized Anxiety Disorder (GAD-7) scale [49]. The participants evaluated the frequency of anxiety symptoms in the last two weeks on a 4-point Likert scale (from *Not at all* = 0, to *Nearly every day* = 3). Higher scores mean greater severity of anxiety. In this study, Cronbach’s α was 0.92.

Depression symptoms were assessed using the 9-item Patient Health Questionnaire (PHQ-9) [50]. Using a 4-point Likert scale (from *Not at all* = 0, to *Nearly every day* = 3), the students rated how often they experienced a given depression symptom in the past two weeks. Higher scores indicate a higher risk of depression. In this study, Cronbach’s α was 0.91.

Life satisfaction was assessed using the 5-item Satisfaction With Life Scale (SWLS) [51]. Using a 7-point Likert scale (from Strongly disagree = 1 to *Strongly agree* = 7), the participants rated how satisfied they are with various aspects of their life. Higher scores are interpreted as greater life satisfaction. The Cronbach’s α in the present sample was 0.88.

Sociodemographic questions were related to age (date of birth), gender (*Women* = 1, *Men* = 0, *Other*), relationship status (*Single* = 1, *In a couple* = 0), faculty of the study (open question), study major (open question), level (*Bachelor’s, Master’s, uniform five-year Master’s, uniform six-year Master’s*), year of study (*from 1 to 6*), and type of study (*full time, part time*).

### 2.3. Participants

The participants in this study were university students (*N* = 706), aged between 18 and 56 years (*M* = 22.88, *SD* = 5.83), including 539 (76%) women, 148 (21%) men, and 19 (2.7%) non-binary people. The single relationship status was found in 254 students, while 452 students identified their status as “in a couple”. Most participants were in their first year (*n* = 252, 49.85%), followed by second year (*n* = 202, 28.61%), third year (*n* = 93, 13.17%), fourth year (*n* = 30, 4.25%), fifth year (*n* = 25, 3.54%), and sixth year (*n* = 4, 0.57%). The majority were full-time students (*n* = 514, 72.80%) rather than part-time students (*n* = 192, 27.20%), and most were studying toward a Bachelor’s degree (*n* = 416, 58.92%), followed by a Master’s degree (*n* = 42, 5.94%), uniform five-year Master’s (*n* = 156, 22.10%), and uniform six-year Master’s (*n* = 92, 13.03%). The students represented almost 30 various fields of study, including medical, health sciences, social sciences, natural sciences, mechanical, engineering, and technological faculties. Table 1 demonstrates the gender, relationship status, and response rates of the respondents to the eight questions about exposure to COVID-19, stratified by vaccination status (vaccinated, unvaccinated).

### 2.4. Statistical Analysis

A preliminary descriptive statistical analysis (i.e., mean, standard deviation, median, skewness, and kurtosis) was conducted to examine the parametric properties of continuous variables, including age, exposure to COVID-19 pandemic, spirituality, perceived physical health, stress, coronavirus-related PTSD, fear of COVID-19, anxiety, depression, and life satisfaction. The differences between the vaccinated and unvaccinated groups were assessed using Pearson’s Χ^2^ independence test for categorical variables (such as gender, relationship status, and the particular dimensions of exposure to the COVID-19 pandemic) and Student’s *t*-test for continuous variables, except age, which was tested using a non-parametric Mann–Whitney *U* test, because of the study sample’s deviation from normal distribution assumption. The effect size was assessed using φ for categorical variables and Cohen’s *d* for continuous.

The associations between variables were examined using the network analysis (NA) method in the vaccinated and unvaccinated groups. Since categorical and continuous variables were included simultaneously in the NA model, we used the extended Bayesian information criteria and graphical least absolute shrinkage and selection operator (EBICglasso) as an estimator to establish the associations between variables with normalized centrality measures. The weighted network shows the magnitude between nodes (thicker lines between nodes indicate a stronger relationship) and the direction of the association (the blue color represents a positive association, while the red color is negative). In addition, closer nodes mean that they have stronger correlations. Centrality indices such as betweenness, closeness, degree, and the expected influence, help examine the relevance of variables in a model and identify the most important variables and their role in the network. Betweenness represents the degree of connectivity, considered as the number of times a node is a part among all the pairs of the associated nodes in the network. Considering closeness, the more central nodes in the network indicate a closer location to other nodes. Degree (or strength) is the sum of all the paths that connect the nodes in terms of the mean of correlation weights. The expected influence shows the most important variables that can act as a bridge between the adjacent nodes. All statistical tests were performed using the JASP ver. 0.16.1.0. software for Windows.

## 3. Results

### 3.1. Differences between the Vaccinated and Unvaccinated Participants in Demographic Variables and Well-Being Dimensions

The first step in the statistical analysis was to check the parametric properties of all continuous variables. Skewness ad kurtosis was in the range of ±1 for all the variables (excluding age), indicating good characteristics for parametric analyses in a large sample size (*N* = 706). Age was examined in both groups of vaccinated and unvaccinated students using a non-parametric Mann–Whitney *U* test. Significantly younger participants were revealed in the sample of unvaccinated (*M* = 22.17, *SD* = 5.78, *Mdn*. = 21) than vaccinated individuals (*M* = 23.07, *SD* = 5.84, *Mdn*. = 21), with *U* = 36401, *p* = 0.019, and Cohen’s *d* = −0.123. The differences between the vaccinated and unvaccinated samples in terms of the categorical variables, such as gender (women vs. men), relationship status (single vs. in a couple), and the eight aspects of exposure to COVID-19, were examined using Pearson’s Χ^2^ test of independence (Table 1). Neither the gender nor the relationship status differentiated between the vaccinated and unvaccinated groups. Significantly more vaccinated participants experienced symptoms of coronavirus infection (exposure 1) and being in strict quarantine for at least 14 days (exposure 4), and this group more frequently had an infected family member or friend (exposure 5) compared with their unvaccinated counterparts.

**Figure 1:**
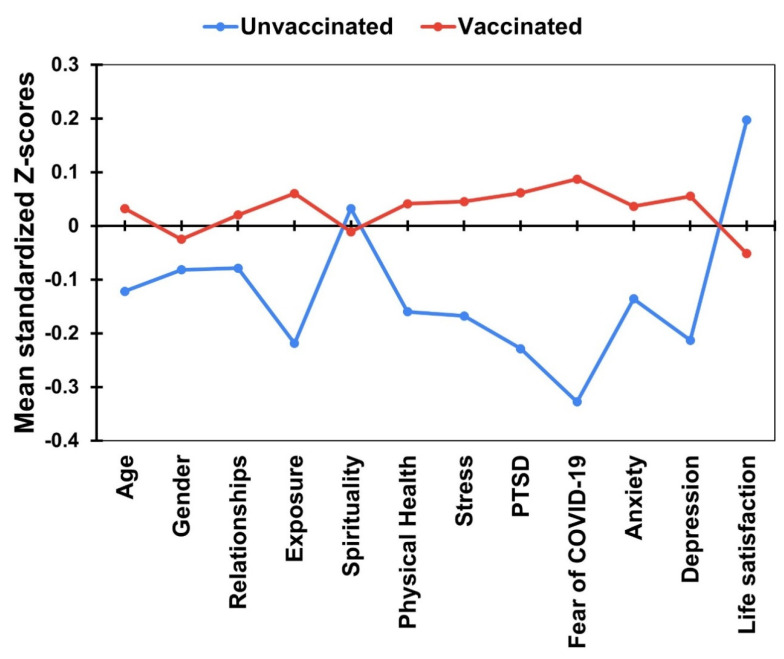
Differences between vaccinated and unvaccinated participants in mean standardized Z-scores of demographic variables and well-being dimensions.

Student’s *t*-test was performed to examine whether vaccination differs between variables such as exposure to the COVID-19 pandemic, perceived physical health, spirituality, perceived stress, coronavirus-related PTSD, fear of COVID-19, anxiety, depression, and life satisfaction. As shown in Table 2, significant differences between vaccinated and unvaccinated university students were found in all the variables, except spirituality and anxiety. Unvaccinated participants presented significantly lower scores than their vaccinated counterparts in exposure to the COVID-19 pandemic, perceived physical health, stress, coronavirus-related PTSD, fear of COVID-19, and depression. In contrast, higher scores were demonstrated in life satisfaction among the unvaccinated individuals than among the sample of vaccinated people. We converted raw scores into standardized *Z* scores to better visualize the group differences relative to all the variables (demographics and well-being dimensions) in one plot, which is presented in Figure 1.

### 3.2. Association between Demographic Variables and Well-Being Dimensions in Vaccinated and Unvaccinated Samples of University Students

**Figure 2:**
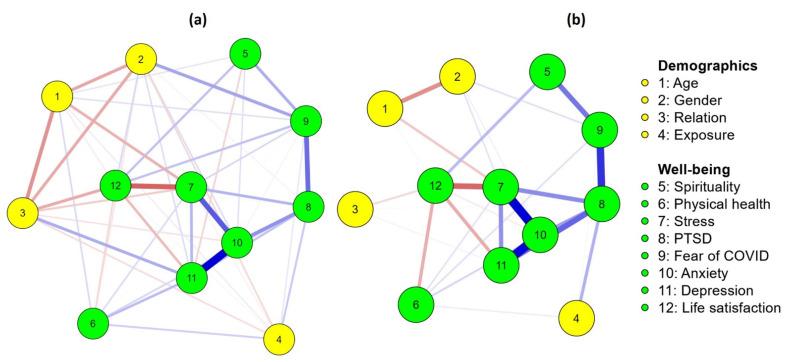
The network structure of demographics and well-being dimensions in the group of (**a**) vaccinated and (**b**) unvaccinated university students during the fourth wave of the COVID-19 pandemic. Red lines represent negative associations, while blue lines represent positive relationships. Line thickness represents the strength of the links. Relation = relationship status; PTSD = coronavirus-related post-traumatic stress disorder. *N* = 706.

**Figure 3:**
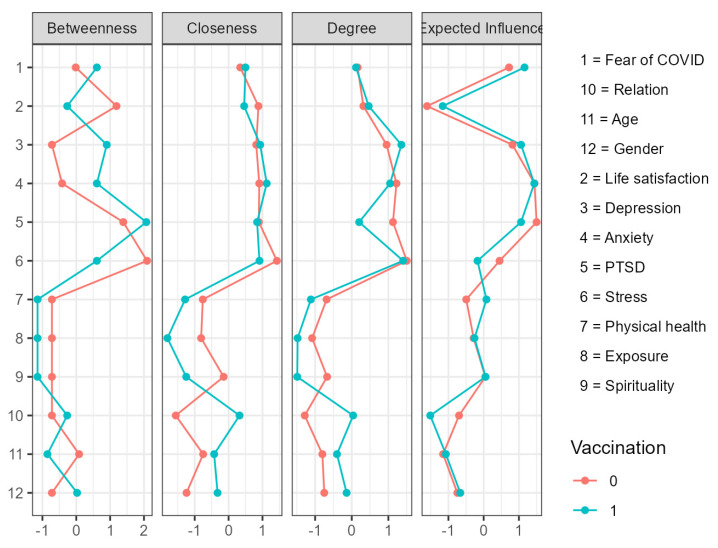
Centrality plot (betweenness, closeness, degree, and expected influence). Standardized Z score values in several demographic variables and well-being dimensions among vaccinated = 0 (red) and unvaccinated = 1 (green) samples of university students during the fourth wave of the COVID-19 pandemic. *N* = 706.

To explore the associations between demographic variables and well-being dimensions, the network analysis (NA) was performed for vaccinated and unvaccinated university students. The results are shown in Figure 2 and Figure 3. In the unvaccinated group, the strongest edges were found between depression–anxiety and anxiety–stress symptoms, with anxiety acting as a bridging symptom for increasing stress and depression. Next, strong and positive correlations were found between fear of COVID-19 and coronavirus-related PTSD, between fear of COVID-19 and spirituality, and between coronavirus-related PTSD and anxiety. Stress was a bridging symptom for increasing anxiety and decreasing life satisfaction. Life satisfaction was inversely related as a bridging variable to physical health and depression. Younger age was related to the female gender in the sample of unvaccinated university students. Exposure to the COVID-19 pandemic was positively associated with coronavirus-related PTSD, while spirituality was positively related to life satisfaction.

Regarding the vaccinated group, more interconnections were presented compared with the unvaccinated participants. The strongest connections were demonstrated between anxiety and depression, followed by a link between anxiety and stress, a weaker positive relationship between fear of COVID-19 and coronavirus-related PTSD, and a negative association between stress and life satisfaction. Anxiety was a bridging symptom for increasing depression, perceived stress, and coronavirus-related PTSD. Younger age was related to both the single status and female gender among the vaccinated students. Moreover, the single relationship status was a bridging variable, which decreased life satisfaction and increased symptoms of depression. Higher fear of COVID was associated with the female gender in the sample of vaccinated individuals.

## 4. Discussion

Although various variables were previously examined as the potential determinants of decision about vaccination, this study explored for the first time a large number of variables related to subjective well-being and quality of life among vaccinated and unvaccinated university students during the fourth wave of the COVID-19 pandemic. Supporting the fuzzy-trace theory approach [40] and our expectations, the vaccinated and unvaccinated participants differed in their physical and mental health and well-being. In particular, the unvaccinated participants showed a significantly younger age, experienced fewer symptoms of coronavirus infection, and were more frequently reported not to be strictly quarantined or to have an infected family or friend compared with their vaccinated counterparts. Furthermore, the unvaccinated students presented lower composite scores in exposure to the COVID-19 pandemic, spirituality, perceived physical health and stress, coronavirus-related PTSD, fear of COVID-19, anxiety, and depression, but a higher level of life satisfaction than the vaccinated sample. We can assume that younger and generally healthy university students avoid vaccination to maintain their status quo of “feeling okay” [40]. This anti-vaccination behavior can be justified in various ways: increasing a subjective sense of vaccination fear, exaggerating possible side effects, or even via irrational explanations, such as believing in conspiracy theories. However, the primary reason for non-vaccination may be maintaining a positive subjective sense of well-being. In contrast, individuals who currently “do not feel okay” have much more motives to accept the risk of vaccination, even if they are concerned about possible side effects similar to the unvaccinated individuals. Participants with poor physical health, high symptoms of stress, coronavirus-related post-traumatic stress disorder (PTSD), anxiety and depression, and low life satisfaction levels may be more willing to receive the vaccine as a means to improve their well-being or not to worsen the situation, maintaining the status quo of “not feeling okay”.

The present study is consistent with some previous research [21,22,27,28,30]. In a study conducted on a large Moroccan sample (*n* = 3800), several factors were considered for the intention to receive the vaccine, including perceived susceptibility and severity, benefits and barriers, self-efficacy, or cues to action [22]. A higher acceptance of the COVID-19 vaccine was related to having a chronic disease. Most Algerian citizens were unwilling to receive a second booster dose of the COVID-19 vaccine (only 13.2% declared receiving), which was associated with the belief in the inefficiency of vaccination and that the primer dose was sufficient [27]. A higher acceptance rate of booster vaccination was found among people with chronic comorbidities and experience of COVID-19 infection. Another study among Japanese university students examined vaccine intention and concerns over the COVID-19 pandemic based on the health belief model (HBM) [28]. Different components of health-related behavior were utilized in the study, such as the subjective perception of susceptibility to COVID infection, the severity of being infected, barriers and benefits of vaccination, and cues to action (e.g., the role of authorities, relatives, media, or government). Most participants (92%) were willing to receive the COVID-19 vaccine, and positive predictors of active intentions were participation in healthcare courses among students, perceived severity of COVID-19 infection, and benefits from HBM. In contrast, the perceived barriers (e.g., side effects, troublesome, and parent disagreement) were negative predictors. Vaccine hesitancy was also explored among healthcare workers in Singapore community hospitals regarding various vaccine-related factors such as perceptions, attitudes, knowledge, and contextual features. Vaccine hesitancy was associated with younger age and not having a loved one or friend infected with COVID-19 [21], which seems consistent with this study. Similarly, a lower vaccination willingness was found among younger coronavirus-infected German participants, those with low income, who do not trust vaccination effectiveness and the governmental pandemic management, those who experience high fear of adverse vaccination side effects, have insufficient knowledge about SARS-CoV-2, and believe in conspiracy theories [26].

However, this study contradicts other studies that demonstrated higher levels of well-being among the vaccinated than unvaccinated people [6,15,23,25]. Galanis et al. [23] investigated the participants’ willingness to receive a second COVID-19 booster dose of vaccine. Individuals unwilling to vaccinate were concerned about the second dose’s side effects and ineffectiveness. The sample of people willing to receive a second COVID-19 booster mainly consisted of younger participants, those without a previous COVID-19 diagnosis, those who perceived physical health as good or very, and those who reported increased fear of the COVID-19 alongside increased trust in COVID-19 vaccinations. Koesnoe et al. [25] explored the intention or acceptability of COVID-19 vaccination among Indonesian healthcare workers based on the health behavior theory and the integrated behavior model (IBM). The predictors of vaccination intention included favorable vaccine attitudes, perceived norms, and self-efficacy. In contrast, women prevailed among people with low vaccination intentions, as well as participants with low income, married individuals, and those with a history of COVID-19 infection.

The “feeling-better” effect can explain the improved well-being among vaccinated people after subsequent vaccination. Some people may strongly believe in the beneficial effects of vaccination or are more susceptible to the placebo effect. Indeed, a recent study showed that vaccination against COVID-19 decreases the level of anxiety and fear of being infected among university students from Poland [15]. A study on a nationally representative sample of US adults (*n* = 8090) showed that perceived risks of infection, hospitalization, and death, as well as psychological distress (assessed by using the four-item Patient Health Questionnaire (PHQ-4) measuring depression symptoms), declined in people vaccinated against COVID-19 [52]. Furthermore, Agrawal et al. [19], in a large sample of the US Census Bureau’s Household Pulse Survey (2,035,847 adults, including 247,406 vaccinated and 1,788,441 unvaccinated), found that anxiety and depression symptoms were significantly reduced, by about 28% and 27%, respectively, among vaccinated participants. In contrast, higher vaccination rates in the population were not associated with fewer symptoms of anxiety or depression in unvaccinated individuals. Therefore, vaccination can not only be used to reduce the risk of coronavirus infection but also as a coping strategy to improve the population’s mental health during the pandemic.

Gender was not related to vaccination in this study. Previous research was inconclusive, showing that women dominated the unvaccinated or undecided group [21,23,25,27,29], whereas in another study, men prevailed in these groups [22]. Tucker et al. [31] showed that vaccination was not related to demographics, mental health, or substance use among the unvaccinated young adults who recently experienced homelessness in Los Angeles (USA). Therefore, more research is necessary to explain gender roles in vaccination. Since gender is a sociocultural variable, the interaction between gender and country may be more predictive of a decision about vaccination. Vaccination acceptancy seems to vary between countries, as indicated by a recent review and meta-analyses [29]. Future studies may examine whether the interaction between gender and country can contribute to vaccination decisions.

For the first time, to our best knowledge, the NA was used to explore specific associations between demographic variables and well-being dimensions among vaccinated and unvaccinated university students. Summarizing the results, there were numerous similarities in network structure in the vaccinated and unvaccinated samples. In both groups, the strongest positive associations were found between symptoms of depression–anxiety and anxiety–stress, with the mediating role of stress in reducing life satisfaction. However, differences were also demonstrated, especially in the higher interconnectedness of well-being dimensions with demographic variables (age, gender, relationship status) and exposure to COVID in the vaccinated sample, compared with the unvaccinated. The most influential variables were successively anxiety, depression, coronavirus-related PTSD, and fear of COVID. However, stress played an essential role in combining the deteriorated mental health symptoms with life satisfaction. Although there were some slight differences between the vaccinated and unvaccinated samples regarding the strengths of interconnections, the overall network pattern was similar in both groups. Further studies can examine the role of health-related behaviors and vaccination since previous research found that people presented rather unhealthy behavior during the pandemic [5].

A quantitative study, based on the established behavioral change technique (BCT) taxonomy, identified five main barriers to receiving vaccination among the general population in Hong Kong: concerns about severe and long-term side effects, the perceived low protection ability of vaccination, unclear information on the program of vaccination, insufficient data on safety and effectiveness, and distrust in safety and effectiveness vaccination [34]. Based on the BCT research and theory, Wong et al. [34] recommended implementing several strategies to increase vaccination acceptance, such as promoting trustworthy vaccine-related information and social influence, emphasizing social responsibility and healthcare professionals’ recommendations, incentives, and increasing accessibility for vaccinations.

### Limitation of the Study

Although the sample size was quite large (*N* = 704), the limitations of this study include its lack of a balanced structure regarding gender (women prevailed), as well as a broad range of age and year of the study (students in the first study year prevailed). Therefore, the results of this study cannot be generalized to the whole adult or student population from other countries. Future studies should be performed in a more representative sample of university students and using experimental or qualitative methods to examine whether their results support those of the present study. Future international surveys may be conducted on different populations regarding employment or student status, ages, and other countries. Additionally, the snowball sampling method and online survey may cause some bias. Self-reported measures were used in this study to assess physical and mental health and well-being, which can also produce some distortions in results. Moreover, most of the standardized questionnaires measured the variables very briefly, using scales with few items. Although a long survey carries a risk of fatigue and test abandonment, brief measures can also become a source of measurement bias. Although the NA shows influential variables, due to the cross-sectional design of this study, the results should be considered with caution. Exposure to the COVID-19 pandemic and spirituality were assessed using a newly developed questionnaire; thus, more studies are necessary to validate these scales and to repeat the current research methodology using scales of known parametric properties. The present result of the exposure and spirituality scales should be carefully handled.

## 5. Conclusions

The results of this study contribute to the existing literature, by providing evidence that the fuzzy-trace theory is useful to explain and predict vaccination behavior during the COVID-19 pandemic. Furthermore, the study showed, for the first time, how particular dimensions of mental health and well-being interplay in vaccinated and unvaccinated people during the global crisis of COVID-19. The results of this study have some implications for preventive medicine and psychology. Since younger and overall healthy university students are less willing to vaccinate than their older counterparts with lower well-being status and worse health, the promotion campaigns at universities should be focused on showing the beneficial well-being effects of vaccination in terms of improving physical and mental health or maintaining the status quo of good health by vaccination. Moreover, information about potential side effects cannot obscure the beneficial effects of vaccination. This information seems to increase fear of vaccination and constitutes a significant barrier for unvaccinated people. Myths about vaccination and irrational or false beliefs (e.g., conspiracy theories) should be refuted based on scientific facts. Reliable epidemiological information about the frequency of side effects and the results of numerous research studies on the beneficial effects of vaccination should be provided at universities for all academic societies. Since healthcare courses increase people’s willingness to vaccinate, university authorities should universally implement such approaches.

The other implication of our findings concerns the results of the NA. Anxiety and stress should be considered targets for improvement among university students since they were found in this study as bridging symptoms for increasing depression and worsening life satisfaction. Universities should offer a variety of courses to students that teach negative emotion, stress management, and anxiety reduction (e.g., coping with stress strategies, mindfulness, relaxation techniques, etc.). These activities can help to prevent depression and increase the subjective well-being of the student population.

## Figures and Tables

**Table 1:** Demographic variables and exposure to COVID-19 in the university students’ sample (*n* = 706).

Variable	Total	Vaccination		χ^2^(1)	*p*	φ
		Unvaccinated (*n* = 149)	Vaccinated (*n* = 557)			
Gender				0.01	0.925	–0.004
Woman	539 (76.34%)	111 (20.59%)	428 (79.41%)			
Man	148 (20.96%)	31 (20.95%)	117 (79.05%)			
Relationship status				1.16	0.281	−0.041
Single	254 (35.98%)	48 (18.90%)	206 (81.10%)			
In a couple	452 (64.02%)	101 (22.35%)	351 (77.65%)			
Exposure 1				6.29	0.012	0.094
No	386 (54.67%)	95 (24.61%)	291 (75.39%)			
Yes	320 (45.33%)	54 (16.88%)	266 (83.12%)			
Exposure 2				2.04	0.182	0.054
No	414 (58.64%)	95 (22.95%)	319 (77.05%)			
Yes	292 (41.36%)	54 (18.49%)	238 (81.51%)			
Exposure 3				0.02 _a_	0.974	0.001
No	682 (96.60%)	144 (21.11%)	538 (78.89%)			
Yes	24 (3.40%)	5 (20.83%)	19 (79.17%)			
Exposure 4				5.43	0.020	0.088
No	547 (77.48%)	126 (23.04%)	421 (76.97%)			
Yes	159 (22.52%)	23 (14.47%)	136 (85.54%)			
Exposure 5				7.24	0.007	0.101
No	177 (25.07%)	50 (28.25%)	127 (71.75%)			
Yes	529 (74.93%)	99 (18.71%)	430 (81.29%)			
Exposure 6				0.67	0.414	0.031
No	581 (82.29%)	126 (21.69%)	455 (78.31%)			
Yes	125 (17.71%)	23 (18.40%)	102 (81.60%)			
Exposure 7				0.41	0.525	0.024
No	565 (80.03%)	122 (21.59%)	443 (78.41%)			
Yes	141 (19.97%)	27 (19.15%)	114 (80.85%)			
Exposure 8				0.55	0.457	0.028
No	341 (48.30%)	76 (22.29%)	265 (77.71%)			
Yes	365 (51.70%)	73 (20.00%)	292 (80.00%)			

Note. Exposure = exposure to COVID-19 to assess the consequences of COVID-19: Exposure 1 = symptoms of coronavirus infection, Exposure 2 = testing for coronavirus, Exposure 3 = hospitalized, Exposure 4 = in strict quarantine for at least 14 days, Exposure 5 = an infected family member or friend, Exposure 6 = death of a loved one or relatives, Exposure 7 = job loss, Exposure 8 = a worsening economic status. a = Fisher’s exact test for small-sample size.

**Table 2:** Student’s *t*-test for comparison of vaccinated and unvaccinated university students relative to well-being dimensions.

Variables	Unvaccinated (*n* = 142)		Vaccinated (*n* = 545)		*t*(704)	*p*	Cohen’s *d*
	*M*	*SD*	*M*	*SD*			
Exposure to COVID-19	2.40	1.59	2.87	1.69	–3.02	0.003	–0.279
Spirituality	6.78	3.34	6.64	3.19	0.46	0.643	0.043
Physical health	4.97	1.72	5.32	1.77	–2.19	0.029	–0.201
Perceived stress	20.50	8.88	22.17	7.55	–2.31	0.021	–0.213
Coronavirus-related PTSD	11.78	4.13	12.98	4.12	–3.16	0.002	–0.292
Fear of COVID	11.38	4.93	13.46	4.97	–4.56	<0.001	–0.42
Anxiety	7.75	5.08	8.66	5.40	–1.87	0.062	–0.172
Depression	8.85	6.05	10.63	6.77	–2.93	0.004	–0.27
Life satisfaction	22.36	6.21	20.76	6.46	2.71	0.007	0.25

## Data Availability

The data are submitted to Mendeley Dataset: Rogowska, Aleksandra (2022), “Mental health and well-being of university students during the fourth wave of the COVID-19 pandemic”, Mendeley Data, V1, doi: 10.17632/bvcwx4tfjm.1. Available on: http://dx.doi.org/10.17632/bvcwx4tfjm.1 (accessed on 16 July 2021).
